# The Antibiotic Kitasamycin—A Potential Agent for Specific Fibrosis Preventing Therapy after Fistulating Glaucoma Surgery?

**DOI:** 10.3390/pharmaceutics15020329

**Published:** 2023-01-18

**Authors:** Katharina A. Sterenczak, Georg Fuellen, Anselm Jünemann, Rudolf F. Guthoff, Oliver Stachs, Thomas Stahnke

**Affiliations:** 1 Department of Ophthalmology, Rostock University Medical Center, 18057 Rostock, Germany; 2 Department of Obstetrics and Gynecology, University of Rostock, 18059 Rostock, Germany; 3 Institute for Biostatistics and Informatics in Medicine and Ageing Research, Rostock University Medical Center, 18057 Rostock, Germany; 4 Department Life, Light & Matter, University of Rostock, 18059 Rostock, Germany; 5 Institute for Implant Technology and Biomaterials e.V., 18119 Rostock, Germany

**Keywords:** glaucoma surgery, fibrosis, kitasamycin, novel pharmaceutical concepts, specific antifibrotic agent

## Abstract

One major complication after fistulating glaucoma surgeries are fibroblast-mediated scarring processes and their specific prevention is key in the development of novel pharmaceutical concepts. Within this study a possible antifibrotic potential of kitasamycin (KM) in a transforming growth factor (TGF)-β1-mediated fibroblast model was evaluated *in vitro*. Primary ocular fibroblasts were isolated, cultivated and a dose–response test including determination of the half maximal effective concentration (EC50) for KM was conducted. Transformation of fibroblasts into myofibroblasts was induced by TGF-β1and immunofluorescence (IF), and Western blot (WB) analyses were performed with fibroblasts and myofibroblasts. IF analyses were carried out using antibodies against α-smooth muscle actin (α-SMA) and fibronectin, and protein detection of intracellular and extracellular proteins was performed by WB. Using the dose–response test, the viability, cytotoxicity and EC50 of KM after 24 and 48 h were determined. Fibroblasts exposed to various KM concentrations showed no increase in α-SMA and extracellular matrix expression. In TGF-ß1-stimulated myofibroblasts, KM inhibited the expression of α-SMA and fibronectin in a concentration-dependent manner. These findings demonstrate that KM could impair the transformation of fibroblasts into myofibroblasts and the expression of proteins involved in fibrotic processes, representing a potential agent for specific fibrosis prevention in future therapeutic concepts.

## 1. Introduction

One of the main causes of irreversible blindness worldwide is glaucoma, which is considered to be a group of diseases that all show a characteristic optic neuropathy as a common endpoint, determined by both structural changes and functional deficits [1]. In addition to age, the main risk factors include, in particular, a genetic predisposition. Thus, the incidence of open-angle glaucoma increases with age and the prevalence of the disease is more common in people of African descent [1]. In contrast, angle-closure glaucoma is a common form of glaucoma in people of Asian descent [1]. In 2013, Tham et al. [2] estimated the number of people with glaucoma worldwide to be approximately 64 million. Furthermore, the number is expected to increase to approximately 112 million by 2040, with people of Asian and African descent being disproportionately affected [2].

Current therapy for the treatment of glaucoma is the reduction in intraocular pressure (IOP). Besides this, there are other therapeutic non-IOP-related interventions such as neuroprotection preventing or delaying glaucomatous neurodegeneration, independently of IOP [3,4,5]. Furthermore, gene and cell therapies, for example, have the potential to provide more effective and durable treatments or even cures by targeting specific disease mechanisms [6]. Although these therapies have not yet received clinical approval, they have shown potential in laboratory trials and various glaucoma animal models [6].

Standard drug therapy for IOP lowering with eye drops is often limited to allergic reactions and dry eye disease caused by preservatives contained in eye drops [7]. The lack of patient adherence and insufficient success with drop therapy necessitate sustained alternative forms of therapy [8,9]. Surgical interventions such as trabeculectomy are used as invasive forms of therapy [10,11]. In 1969, Molteno et al. [12] introduced glaucoma drainage devices which have been increasingly used in glaucoma surgery ever since. Recently, promising new devices for microinvasive glaucoma surgery (MIGS) have been developed as alternatives to the conventional surgical interventions, minimizing complications such as implantation-induced ocular traumas and lesions [13,14]. In the majority of fistulating glaucoma surgeries, Tenon’s space is used as the outflow area for aqueous humor drainage from the eye into a reservoir called a “bleb”. Tenon’s space is a loose connective tissue layer between the conjunctiva and sclera and serves as a displacement layer. This loose connective tissue space is characterized by the fact that it can be expanded as well as absorb large amounts of fluid. Tenon’s fibroblasts are responsible for the physiological maintenance of this tissue structure. A complication of draining surgical techniques is a scarring reaction in Tenon’s space, which is characterized by the transformation of Tenon’s fibroblasts into myofibroblasts. Long-term results of these invasive therapies show that fibrotic processes and uncontrolled, excessive and persistent scarring lead to a decrease in aqueous humor outflow, which in turn results in an increase in IOP [10,11,15,16]. To prevent fibroblast-mediated fibrosis, cytostatic agents such as mitomycin C (MMC) can be used, but they are associated with side effects such as an avascular, infected or rupturing bleb [15,17]. In this regard, MMC acts non-specifically by irreversibly intercalating between the DNA double strands of cells, thereby preventing cellular processes such as replication or transcription, ultimately leading to cell death [18]. In Tenon’s fibroblast cultures, MMC induces apoptosis, which explains its long-lasting antiproliferative effect even after a single administration during trabeculectomy [17]. Thus, a key aspect in the development of advanced therapies could be the specific prevention of fibrotic processes by the targeted inhibition of fibroblasts. There is a great need for more effective therapies that target specific disease mechanisms to prevent progressive neuropathy.

Recently, the macrolide antibiotic josamycin (JM) was identified as a fibrosis-counteracting agent using bioinformatics analysis based on the connectivity map database (CMap) for drug repositioning [19,20]. This was subsequently evaluated in a cell culture model *in vitro*, in which the addition of TGF-β1 induced a fibrosis-like state and the transformation of fibroblasts to myofibroblasts. It was demonstrated that JM inhibited the transformation of fibroblasts to myofibroblasts [19]. In addition, a decrease in the synthesis of extracellular matrix (ECM) components such as fibronectin and collagen was observed. JM and kitasamycin (KM) investigated in this study represent a family of a total of 14 macrolide antibiotics, which differ within this family by their acyl substituent of the mycarose moiety and by the alternation of a hydroxyl and an acetyl group in their 16-membered lactone [21]. In general KM’s mode of action is to inhibit the protein synthesis process. Its inhibition spectrum includes Mycoplasma, Gram-positive bacteria, some Gram-negative bacteria, Leptospira and Rickettsia. It also inhibits most bacteria resistant to penicillin, oxytetracycline, chlortetracycline, erythromycin and chloramphenicol bacteria strains. It is a safe and high-efficacy growth-promoting additive for swine and poultry [22]. In the clinical setting, KM is used, among other indications, for inflammatory respiratory diseases similar to JM [23,24,25].

The question arose whether KM, with a similar structure as JM, has a similar biological effect and, therefore, besides antibiotic, also antifibrotic properties. Thus, based on previous work [19], the antifibrotic potential of the antibiotic KM was evaluated in a TGF-β1-mediated human fibroblast model system *in vitro*.

## 2. Materials and Methods

### 2.1. Cell Culture

The present study was approved by the Ethics Committee of the University of Rostock (Approval ID: A 2011 11) and followed the guidelines of the Declaration of Helsinki. Analogous to [26], primary cultures of human Tenon’s fibroblasts (hTFs) were prepared and cultured from tissue samples taken from children during strabismus surgery (Department of Ophthalmology, University of Rostock, Germany) after obtaining written informed consent. For this purpose, the tissue was cut into approximately 1 × 1 mm pieces, placed in 12-well culture plates in DMEM containing 50 U/mL penicillin, 50 mg/mL streptomycin and 10% FCS and incubated at 37 °C in a humidified (95%) incubator under 5% CO_2_. The growth medium was changed three times per week. When the outgrowing primary fibroblasts had formed a confluent single layer, they were trypsinized in 0.25% trypsin/EDTA solution in phosphate-buffered saline (PBS) and subcultured in 25 cm^2^ cell culture flasks.

For the immunofluorescence analyses, cells were seeded and cultured on 12 mm glass coverslips (PAA, Cölbe, Germany) in 12-well plates and for Western blot analysis in 25 cm^2^ cell culture flasks until 60–70% confluence was achieved. Fibroblasts from passages three to five were used for all subsequent analyses. To convert the hTFs to a fibrotic state, they were cultured for 24 h under serum-free conditions and the transformation of fibroblasts to myofibroblasts as well as the expression of fibrotic marker proteins were stimulated by the subsequent addition of 10 ng/mL TGF-β1 for 48 h (11343160; ImmunoTools, Friesoythe, Germany). In this regard, the following experimental test sets were performed and incubated for 48 h each: incubation with KM (1 to 100 µM); incubation with TGF-β1 (10 ng/mL); and incubation with TGF-β1 (10 ng/mL) and KM (1 to 100 µM). For the KM working solution, a 10 mM KM (S3645; SellekChem, Munich, Germany) stock solution was prepared in 99% ethanol (10 mg kitasamycin in 1.2723 mL ethanol) and diluted to the desired concentrations with cell culture medium. As a control, comparable amounts of ethanol without KM in culture medium were applied to the control cells. Experimental approaches and controls were set up for both the immunofluorescence and Western blot analyses and each was performed with 3 biological samples.

### 2.2. Dose–Response Testing of Kitasamycin

Cell-based multiplexed luminescence and fluorescence real-time assays (RealTime-Glo ™ MT, CellTox ™ Green Cytotoxicity; Promega, Madison, WI, USA [16,17]) were used to measure viability (luminescence) and cytotoxicity (fluorescence) using the GloMax^®^ Explorer Multimode Microplate Reader (Promega, Madison, WI, USA). For this purpose, hTF cells were seeded and cultured in 96-well plates (353376; Corning, New York, NY, USA). To determine the linear range and sensitivity of both assays, a linearity assay with different hTF cell densities between 19–20,000 cells/well was performed in advance. For this purpose, the specific cell numbers/well were seeded in 96-well plates. The cell count was performed manually with the Neubauer counting chamber. The linearity test determined 2500 cells/well as a suitable cell density for further measurements (results not shown here). For the dose–response assay, different KM concentrations were used in ascending order (10, 25, 50, 75, 100, 125, 150, 175, 200, 300, 400 and 500 µM KM) on 2500 hTF cells/well. Each concentration was measured fourfold. Measurements were performed at regular intervals from 1 to 48 h after KM addition. Data were analyzed using GloMax Multi Detection System software (version 1.3.2; Promega, Madison, WI, USA). The formula used for EC50/IC50 determination by the instrument´s software was:

(1) $$f\left(x\right)=B+\frac{T-B}{1+10^{(\log\mathrm{IC}50-\log x)S}}$$

(T: Top and B: Bottom Fluorescence/Luminescence; S: Slope)

### 2.3. Evaluation of the Antifibrotic Effect of Kitasamycin

#### 2.3.1. Immunofluorescence

The hTF cells were cultured as described in Section 2.1 and incubated depending on the experimental setup. After the incubation period, cells were washed with PBS and fixed with 3% paraformaldehyde (PFA) for 10 min, followed by three PBS washing steps and incubation with the primary antibodies against α-smooth muscle actin (α-SMA) and fibronectin (ab7817, ab2413; Abcam, Cambridge, UK) at a dilution of 1:100 each. Cells were then washed (3×) with PBS followed by a 45 min incubation with secondary antibodies (711-165-152 (dil. 1:100), 715-545-151 (dil. 1:50); Dianova GmbH, Hamburg, Germany). After incubation with secondary antibodies, cells were again washed (3×) with PBS and finally preserved with embedding medium (Vectashield; Vector Laboratories, Peterborough, UK). Cell nuclei were stained with 4,6-diamidino-2-phenylindole (DAPI) (1 μg/mL) contained in the embedding medium. Fluorescent labeling was analyzed using an Axioskop 40 (Zeiss, Jena, Germany) equipped with an HBO 50 microscope light (Zeiss), Colibri 7 LED solid-state light source (Zeiss), and AxioVision software (version 4.8.1.; Zeiss).

#### 2.3.2. Western Blot Analysis

The hTF cells were cultured as described in Section 2.1 and incubated depending on the experimental setup. For Western blot analysis, primary hTF cells were then washed with PBS and lysed in Laemmli buffer containing 1% SDS. Denaturation was performed at 95 °C for 10 min, and electrophoretic separation (10 to 30 µg proteins/lane) was performed in 7.5% or 10% SDS gels. The transfer was performed on nitrocellulose membranes (0.2 μm; Bio-Rad, Munich, Germany). The membranes were further saturated with 5% nonfat dry milk powder dissolved in TRIS-buffered saline (TBS) for 30 min followed by incubation with the primary antibodies at 4 °C overnight. The following primary antibodies were used (dilutions in parentheses): anti-fibronectin (ab2413 (1:500); Abcam), anti-collagen VI (ab182744 (1:500); Abcam), anti-vimentin (V2258 (1:500); Sigma-Aldrich, St. Louis, MO, USA), anti-α-SMA (ab7817 (1: 500); Abcam), anti-β-actin (A2228 (1:500); Sigma-Aldrich) and anti-β-tubulin (T5293 (1:250); Sigma-Aldrich). Subsequently, membranes were washed (3×) with TBS spiked with Tween 20 (0.1%) and further incubated with secondary anti-mouse or anti-rabbit HRP-conjugated antibodies (NXA931 (1:2500); Amersham, Buckinghamshire, UK; 170–6515 (1:2500); Bio-Rad, Munich, Germany). Visualization of bound antibodies was performed by the enhanced chemiluminescence (ECL) method according to the manufacturer’s protocol (Thermo Fisher Scientific, Pierce, Rockford, USA). The relative quantification of the protein bands was performed with ImageJ software, which is a Java-based freeware by Wayne Rasband from the National Institutes of Health “https://imagej.nih.gov/ij/ (accessed on 14 May 2022)”. Statistical analysis was performed by t-test analysis (two-sample t-test). Untreated fibroblasts (hTFs) on one hand and TGF-β1-stimulated myofibroblasts (hTFs + TGF-β1) on the other hand were used to compare with all other culture approaches. A *p*-value of ≤ 0.05 was considered statistically significant. 

## 3. Results

### 3.1. Dose–Response Testing after In Vitro Incubation of hTFs with Kitasamycin

The live reporter viability assay determined the redox potential of cells in real time, and the signal directly correlated with the number of living, metabolically active cells at the time of measurement [27]. The principle underlying the assay is the measurement of ATP content in an ATP-dependent reaction. The live reporter assay measures cytotoxicity in real time based on binding with the DNA of dead cells [28]. Living cells with intact cell membranes did not contribute to the signal, as the applied cytotoxicity assay (see Section 2.2) is not membrane-permeable; thus, the measured signal was directly proportional to cytotoxicity [28].

#### 3.1.1. Viability

**Figure 1:**
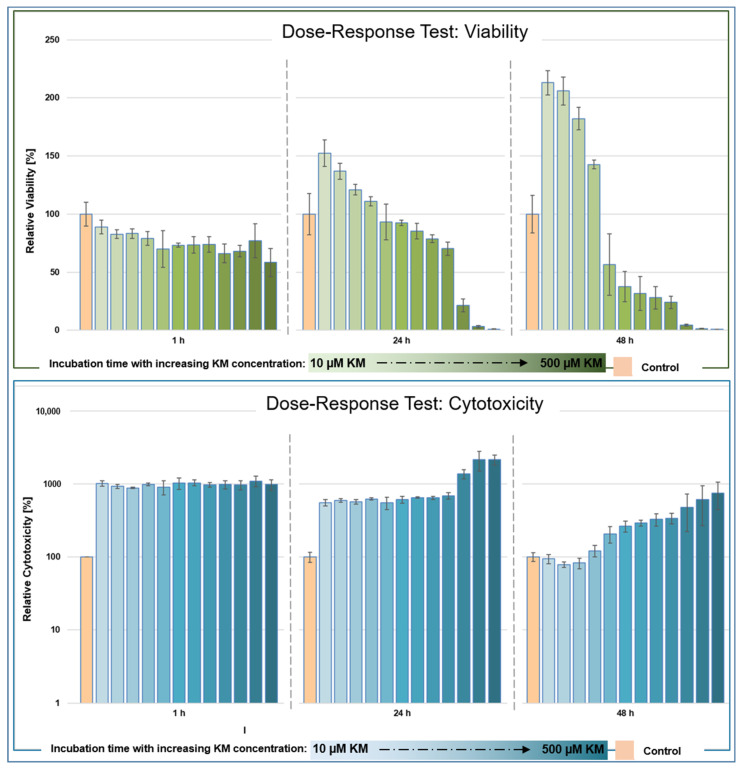
Dose–response test of kitasamycin (KM) in human Tenon’s fibroblasts (hTFs). Cell-based multiplexed luminescence and fluorescence real-time assays were used to measure viability and cytotoxicity. The hTF cells (2500 cells/well) were seeded in a 96-well plate and incubated with different KM concentrations (hTFs + 10, 25, 50, 75, 100, 125, 150, 175, 200, 300, 400 and 500 µM KM) for a total of 48 h. Untreated hTFs served as the control. Each concentration was measured fourfold. Viability measurements (top) and cytotoxicity measurements (bottom) were performed at regular intervals after KM addition. Left: measurement after 1 h; middle: measurement after 24 h; right: measurement after 48 h. The values were normalized to untreated hTFs.

After 1 h of incubation of the hTFs with KM, a concentration-dependent decrease in viability could be measured (Figure 1, top, left). The maximum value was 89% (incubation with 10 µM KM) and the minimum value was 58% (incubation with 500 µM KM). After 24 h of incubation (Figure 1, top, middle), the maximum value was 153% (incubation with 10 µM KM) and the minimum value was 1% (incubation with 500 µM KM). Cells incubated in the range between 10 and 75 µM KM showed higher viability values compared to the controls (between 153% to 111%) and concentrations ≥100 µM showed lower viability values compared to the controls. A decrease in viability to 50% was measured at a concentration of 275 µM KM. After 48 h of incubation (Figure 1, top, right), the maximum value was 213% (incubation with 10 µM KM) and the minimum value was 0.9% (incubation with 500 µM KM). Cells incubated in the range between 10 and 75 µM KM showed higher viability values (213% to 143%) compared to the controls. Concentrations ≥100 µM KM showed lower viability values compared to the control. The half-maximal value (EC50) was reached at 51% when incubated with 100 µM KM.

#### 3.1.2. Cytotoxicity

Incubation of hTFs with KM showed an increase in cytotoxicity levels after 1 h compared to the control (Figure 1, bottom, left). The highest value was 1120% (250 µM KM) and the lowest was 866% (275 µM KM). After 24 h incubation (Figure 1, bottom, middle), the highest cytotoxicity value was 2145% (400 µM) and the lowest was 553% (10 µM kitasamycin), showing a concentration-dependent trend. After 48 h of incubation (Figure 1, bottom, right), the cytotoxicity values decreased and distributed in a concentration-dependent manner in the range between 750% (500 µM) to 94% (10 µM KM) compared to the control. In the concentration range between 10 and 50 µM KM, the cytotoxicity values were below the control level.

### 3.2. In Vitro Evaluation of the Antifibrotic Effect of Kitasamycin in TGF-β1-Mediated Fibrotic Cell Culture Model

Analogous to [13], the immunofluorescence experiments in this study focused on the fibrosis markers fibronectin and α-SMA, whereas the Western blot analyses included α-SMA and fibronectin as well as other intracellular proteins and proteins of the ECM.

#### 3.2.1. Immunofluorescence

**Figure 2:**
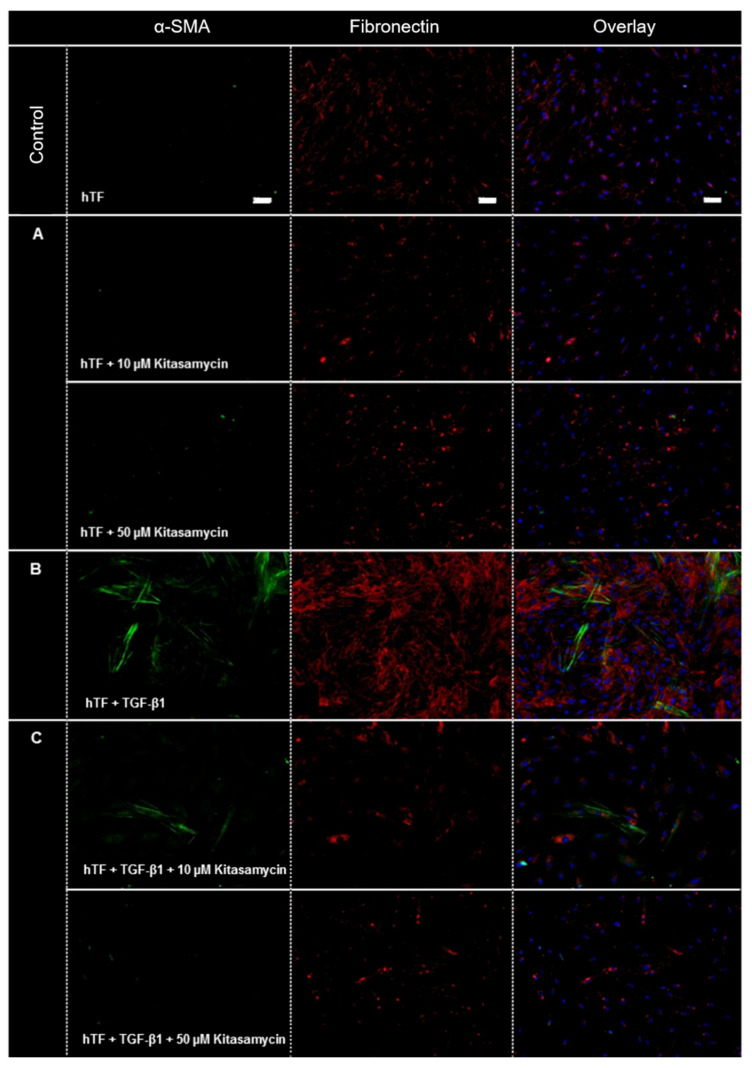
Immunofluorescence-based *in vitro* evaluation of the antifibrotic effect of kitasamycin (KM) in a TGF-β1-mediated fibrotic cell culture model. Immunofluorescence images of α-SMA (green) and fibronectin (red) in human Tenon’s fibroblasts (hTFs) treated with KM (**A**) (hTFs + 10 and 50 µM KM), (**B**) TGF-β1 (hTFs + TGF-β1) and (**C**) the combination of TGF-β1 and KM (hTFs + TGF-β1 + 10 and 50 µM KM) for 48 h. Untreated hTFs served as controls. Cell nuclei were stained with DAPI (blue). Measurement bar: 50 µm.

Figure 2 demonstrates immunofluorescence images of hTFs under the different culture conditions. Under control conditions, expression of fibronectin (red) but no expression of α-SMA (green) was detected (Figure 2). Incubation of hTFs with KM (hTFs + 10 and 50 µM KM) (Figure 2A) showed a similar expression pattern as the control approach. The hTFs incubated with TGF-β1 (Figure 2B) showed a significantly increased expression of fibronectin. Furthermore, this culture condition resulted in the expression of α-SMA in many cells. Cells incubated with the combination of TGF-β1 and KM (hTFs + TGF-β1 + 10 and 50 µM KM) (Figure 2C) demonstrate the regulation of fibronectin expression to the control levels and inhibition of α-SMA expression.

#### 3.2.2. Western Blot Analysis

**Figure 3:**
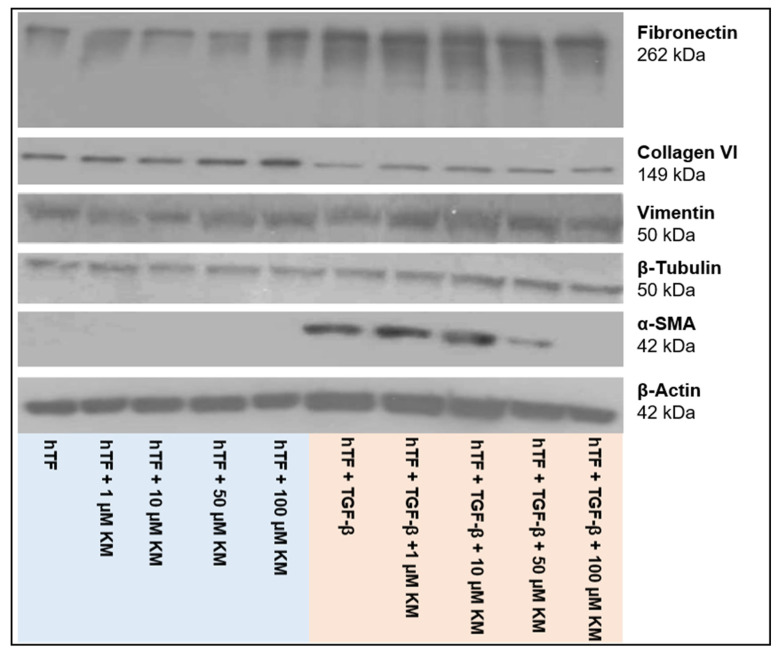
Western blot analysis for *in vitro* evaluation of the antifibrotic effect of kitasamycin (KM) in a TGF-β1-mediated fibrotic cell culture model. Western blot analysis of human Tenon’s fibroblast (hTF) cell lysates under different culture conditions. Cells were treated with increasing concentrations of KM (hTFs + 1–100 µM KM) alone, TGF-β1 alone (hTFs + TGF-β1) and TGF-β1 + KM (hTFs + TGF-β1 + 1–100 µM KM) for 48 h each. Cell lysates of untreated hTFs served as the controls. Antibodies against fibronectin (262 kDa), collagen VI (130 kDa), vimentin (58 kDa), β-tubulin (50 kDa), α-SMA (42 kDa) and β-actin (42 kDa) were used. The β-tubulin served as the loading control.

**Figure 4:**
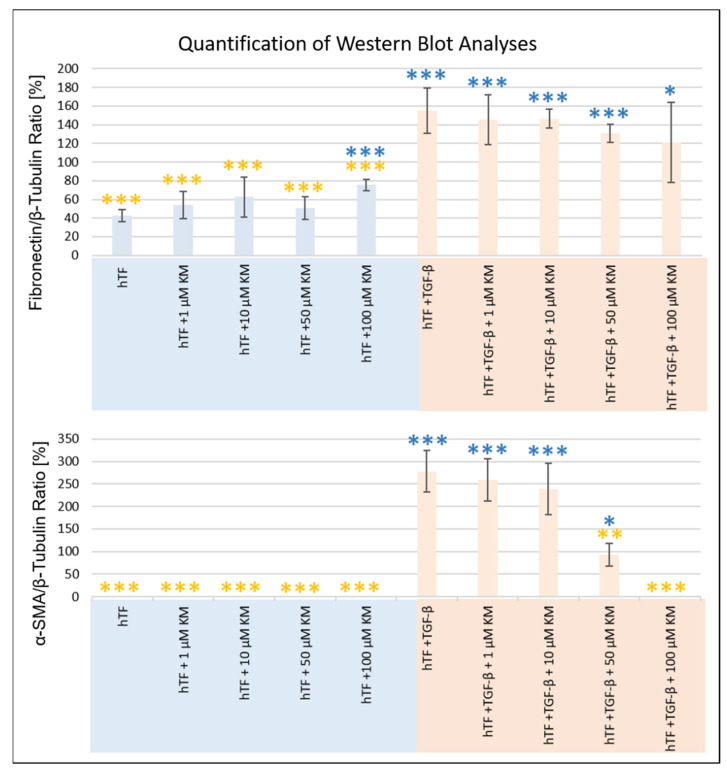
Quantification of Western blot analyses for the *in vitro* evaluation of the antifibrotic effect of kitasamycin (KM) in a TGF-β1-mediated fibrotic cell culture model. Quantification of data from Western blot analysis of human Tenon’s fibroblast (hTF) cell lysates under different culture conditions (Figure 3) for fibronectin and α-SMA. Each column represents a mean ± SD of three experiments. In each case, the values were normalized to the loading control β-tubulin. Statistical analysis was performed using a t-test. The blue asterisk * symbolizes the significance of each approach compared with untreated fibroblasts (hTFs). The orange asterisk * symbolizes the significance of the individual approaches compared with myofibroblasts (hTFs + TGF-β1). A *p*-value of ≤0.05 was considered statistically significant. Significance levels: * *p* ≤ 0.5; ** *p* ≤ 0.01; *** *p* ≤ 0.001.

Figure 3 depicts the expression of the studied intracellular proteins, ECM components and α-SMA at the protein level. Meanwhile, β-tubulin served as a loading control (Figure 3). Intensity determination of the bands was performed using ImageJ software. Normalization of the band intensities was performed using the corresponding β-tubulin band of each approach. Table 1 summarizes the t-test analyses for all proteins studied. Figure 4 presents the normalized values and t-test analyses (fibroblast (hTF) vs. culture approach; myofibroblast (hTF + TGF-β1) vs. culture approach) for fibronectin and α-SMA. 

Fibronectin was expressed in all approaches, with higher band intensities detected in TGF-β1-stimulated myofibroblasts (hTFs + TGF-β1; hTFs + TGF-β1 + 1–100 µM KM) (Figure 3 and Figure 4). The comparison of fibronectin expression between the control fibroblasts (hTFs) and the experimental approaches with 1–50 µM KM-treated fibroblasts was not statistically significant (hTFs + 1–50 µM KM) (Figure 3 and Figure 4, Table 1). Treatment of fibroblasts with 100 µM KM (hTFs + 100 µM KM) showed significantly higher fibronectin expression compared to the control (hTF) (*p* = 0.000) (Figure 3 and Figure 4, Table 1). TGF-β1 stimulated myofibroblasts (hTFs + TGF-β1) and the combination approaches with stimulated myofibroblasts and KM (hTFs + TGF-β1+ 1– 100 µM KM) showed significantly increased fibronectin expression compared to control fibroblasts (hTFs) (*p* = 0.000; *p* = 0.000; *p* = 0.000; *p* = 0.001; *p* = 0.050) (Figure 3 and Figure 4, Table 1). In the combination approach with the highest KM concentration (hTF + TFG-β1 + 100 µM KM), fibronectin expression was decreased and showed a lower significance level compared to the other experimental approaches stimulated with TGF-β1 (*p* = 0.050) (Figure 3 and Figure 4, Table 1). The t-test analysis also performed between TGF-β1-stimulated myofibroblasts and the experimental approaches showed significantly lower expression of fibronectin in the fibroblasts and KM-treated fibroblasts (hTFs; hTFs + 1–100 µM KM) (*p* = 0.000) and no significant differences in KM-treated myofibroblasts (hTFs + TGF-β1 + 1–100 µM KM) (Figure 3 and Figure 4, Table 1). 

Expression of α-SMA was detected in TGF-β1-stimulated myofibroblasts (hTFs + TGF-β1; hTFs + TGF-β1 + 1–50 µM KM) (Figure 3 and Figure 4). The band intensity showed a KM concentration-dependent trend. In the combination approach with the highest KM concentration (hTF + TGF-β1 + 100 µM KM), no α-SMA expression could be detected or α-SMA expression could be totally suppressed by 100 µM KM (Figure 3 and Figure 4, Table 1). Comparison of α-SMA expression between the control fibroblasts (hTFs) and the TGF-β1-stimulated myofibroblasts (hTFs + TGF-β1; hTFs + TGF-β1 + 1–50 µM KM) showed significantly increased α-SMA expression (*p* = 0.000; *p* = 0.000; *p* = 0.000; *p* = 0.035) (Figure 3 and Figure 4, Table 1). In the combination approach with the highest KM concentration (hTF + TFG-β1 + 100 µM KM), α-SMA could not be detected or α-SMA expression could be suppressed by 100 µM KM (Figure 3 and Figure 4, Table 1). The t-test analysis between myofibroblasts (hTFs + TGF-β1) and fibroblasts (hTFs) or KM-treated fibroblasts (hTFs + 1–100 µM KM), which was also performed, showed a significant difference in expression level (*p* = 0.000; *p* = 0.000; *p* = 0.000; *p* = 0.000; *p* = 0.000) (Figure 3 and Figure 4, Table 1). The t-test analysis between myofibroblasts (hTFs + TGF-β1) and KM-treated myofibroblasts (hTFs + TGF-β1 + 1–100 µM KM) showed a KM concentration-dependent trend of α-SMA expression (Figure 3 and Figure 4, Table 1). While no significant difference in α-SMA expression was detected in the approaches with KM concentrations between 1 and 10 µM (hTF + TGF-β1 + 1µM KM; hTF + TGF-β1 + 10 µM KM), the significance level increased at 50 and 100 µM KM (hTF + TGF-β1 + 50 µM KM; hTF + TGF-β1 + 50 µM KM) (*p* = 0.001; *p* = 0.000), suggesting a dose-dependent effect of KM on α-SMA expression (Figure 3 and Figure 4, Table 1).

The expression values of collagen VI, vimentin and β-actin examined here showed variations within the approaches (Figure 3, Table 1). However, no KM-induced mediated antifibrotic effect could be shown within the KM concentrations used here.

## 4. Discussion

Recently, some macrolides have been reported in the literature to exhibit anti-inflammatory, anti-fibrotic and other therapeutic activities in mammalian cells, in addition to their actual antibacterial activity [23,29]. Hence, there is a growing interest in better characterizing the therapeutic effects of macrolides in mammalian cells to guide new applications in both cellular and clinical studies. In our own work, the macrolide JM was identified and evaluated as an antifibrotic agent and evaluated *in vitro* [19,20]. Based on these results, within this study, the antifibrotic potential of macrolide KM was evaluated in a TGF-β1-mediated fibrotic human fibroblast model system *in vitro*. 

The antibacterial mechanism of action of macrolides is thought to be based on the inhibition of protein biosynthesis through reversible binding to the 50S subunit of the bacterial ribosome [30]. Gupta et al. [31] performed a genome-wide shRNA screen in K562 cancer cells to better understand the mode of action of macrolides such as erythromycin and JM in mammalian cells and to identify genes affected by them. Proteins involved in mitochondrial translation, the mitochondrial unfolded protein response, glycolysis and the mitogen-activated protein kinase signaling cascade were identified. Given the similarities between the bacterial and mitochondrial ribosomes, according to [31], the mitochondrial ribosome represents a direct binding target of JM, which is at least partially responsible for the effects on mitochondrial function. Due to the similar structure of KM compared to JM, the mitochondrial ribosome may also represent a direct binding target for KM with potentially comparable effects on affected cells.

Within the present study, a dose–response assay was performed and the mean effective concentration (EC50) of KM in hTFs was determined. The dose–response test showed a decrease in viability as well as an increase in cytotoxicity over the incubation period depending on the KM concentration used. Interestingly, hTFs treated with KM concentrations ≤75 µM showed a different behavior compared to fibroblasts with higher KM concentrations as well as compared to the control hTFs. In these approaches, viability values up to twice as high could be measured. The question arose as to what mechanisms underlie this phenomenon. A pro-proliferative effect of KM could be excluded, as the corresponding cytotoxicity values were at the control level or slightly lower, indicating similarly high cell numbers within the approaches compared to the control. Gupta et al. [31] investigated the oxygen consumption rate of K562 cancer cells after incubation with 15 µM of JM. It was shown that basal and maximal respiration were significantly inhibited by JM through a marked decrease in the total ATP production rate attributable to oxidative phosphorylation [31]. However, in addition, JM-treated cells showed a significant increase in the ATP production rate of glycolysis, with the percentage of total ATP production rate also increasing sharply from 38% to 59%, indicating a significant JM-induced shift in cellular metabolism toward glycolysis [31]. Since cancer cells are highly dependent on glycolysis, the research group also investigated the effects of JM in primary human umbilical vein endothelial cells [31]. The results showed that JM altered cellular energy production in both cancer and primary cells [31]. Due to the related structure, KM may have induced cellular effects similar to those described in [31] in the present study. The increased viability values at low KM concentrations compared to the control levels could also have been caused by a KM-mediated increase in the ATP production rate and a shift in metabolism toward glycolysis. The viability assay used here was based on the measurement of ATP content in an ATP-dependent reaction. Thus, a KM-induced increase in ATP content would be directly reflected proportionally in the measured viability values.

Based on the dose–response results, the evaluation of the antifibrotic effect of KM was then performed analogously to JM [19,20]. Based on the obtained results of the dose–response tests, KM concentrations ranging from 1 to 100 µM KM were used, since higher viability values than the control were detected using these concentrations after 24 h of incubation. The question arose whether these KM concentrations, which are far below the EC50 concentrations, already have an antifibrotic effect. Immunofluorescence and Western blot analyses revealed a different expression behavior of the cells depending on the addition of KM or TGF-β1 alone as well as the combination of TGF-β1 and KM. 

In general, the growth factor TGF-β occupies a key position during wound healing such as after glaucoma filtration surgery [17,32,33] and has been previously shown to be expressed in primary human ocular fibroblast subpopulations [26]. TGF-β is a multifunctional growth factor that is naturally detectable in the aqueous humor of the eye [34]. In this regard, members of the TGF-β family regulate a wide range of cellular functions, and TGF-β-induced processes can be detected in all of the different phases of wound healing: stimulation of fibroblasts and macrophage migration; fibroblast proliferation; angiogenesis; collagen synthesis; modulation of proteolytic enzyme synthesis; secretion of elastin, fibronectin and proteoglycans; and formation of ECM proteins [34]. In this process, activated fibroblasts differentiate into migratory myofibroblasts [15]. One of the main features of this myofibroblastic differentiation is the expression of α-SMA and its organization into stress fibers, which mediate the contractile function of myofibroblasts necessary for wound closure and also play a crucial role in migration to the wound. Within this study, no α-SMA expression was detected in hTFs under control conditions and by the addition of KM alone. Only a cytokine-mediated stimulus by TGF-β1 application induced the differentiation of fibroblasts into myofibroblasts and their synthesis of α-SMA. The organization of α-SMA in stress fibers was demonstrated by immunofluorescence. KM was able to counteract and decrease or suppress the increased expression level of α-SMA-induced by TGF-β1 in a concentration-dependent manner in the combination approaches. 

Furthermore, myofibroblasts are characterized by an increased expression of ECM proteins such as fibronectin and isoforms of the collagen family that are secreted into their extracellular milieu [15,32,35,36]. In this process, myofibroblasts form adhesion complexes at their cell surfaces to bridge the internal microfilaments with extracellular fibronectin domains to create a contractile mechanism that allows these cells to migrate and exert physical influence on their environment [37]. This process is further enhanced by the deposition of collagens [37]. The expression of fibronectin was detected in all experimental approaches. The addition of TGF-β1 initiated an increase in the fibronectin expression rate, which could be decreased in a concentration-dependent manner by the simultaneous application of KM. Another feature of myofibroblastic differentiation is the involvement of extracellular vimentin, which is also a mediator of cell migration and wound repair [35]. However, the results of this study failed to demonstrate higher vimentin expression in myofibroblasts compared to fibroblasts. Vimentin expression was detected in all approaches and showed minor irregularities, but these were not suggestive of a KM-mediated antifibrotic effect in the context of the concentrations used here. 

Furthermore, myofibroblasts are characterized by their increased synthesis of extracellular matrix proteins such as collagens, which are secreted into the extracellular milieu [35]. Collagen VI expression was detected in all approaches and also showed variations in expression levels. However, within these experiments, KM was not able to counteract collagen expression with the concentrations used.

Overall, the *in vitro* analyses performed here cannot be directly transferred to the *in vivo* situation. However, the results obtained provide a first indication of the highest possible applicable concentration as well as the antifibrotic effect of KM for further *in vitro* studies as well as for the first preclinical *in vivo* studies. In the future, within further in vitro experiments as well as a first *in vivo* study, the concentrations around the EC50 value will be investigated. 

A trend of modern therapeutic approaches is the development of “drug depots” which should guarantee a controlled release of therapeutically effective doses within a defined period of time and could inhibit postoperative fibrosis after fistulating glaucoma surgery. These innovative therapy concepts are often based on biocompatible and temporally degrading polymers. The advantage of drug depots in ophthalmology is that the active ingredient is released directly at the site of action (i.e., the eye), minimizing systemic side effects. The results of the present study could provide important information for putative KM concentrations for the establishment of a drug release approach. A KM dosage high enough to impair the transformation of fibroblasts to myofibroblasts but not leading to fibroblast death would be key for drug formulation and the prevention of excessive fibrotic reactions. Furthermore, the transfer of knowledge to other fields of application is conceivable, in which both the antibiotic, anti-inflammatory and antifibrotic properties of macrolide antibiotics may represent an ideal therapeutic option, as already described for JM in upper respiratory tract infections [23]. In further studies, a comparison of the antifibrotic potential of both macrolides (JM vs. KM) would help to identify the most promising candidate in order to achieve a maximum specific antifibrotic effect with minimal side effects in *in vivo* experiments. Therapy with macrolides such as KM and JM could ultimately reduce the severe side effects of cytostatic drugs which are currently used for fibrosis prevention.

## 5. Conclusions

Our study was able to demonstrate a dose-dependent inhibition of expression of fibrotic marker proteins in hTFs by the drug kitasamycin. KM was able to suppress the TGF-β1-induced transformation of fibroblasts into myofibroblasts as well as the expression of proteins involved in scarring processes. The synthesis of fibrotic marker proteins such as α-SMA could be inhibited in a dose-dependent manner and fibrosis-related extracellular marker proteins such as fibronectin could be decreased. Thus, KM represents a potential agent for the specific modulation of postoperative scarring processes and prevention of fibrosis after fistulating glaucoma surgery.

## 6. Patents

K.A.S., G.F., A.J., O.S. and T.S. are listed as inventors in a patent application for the use of josamycin and kitasamycin as antifibrotic agents, filed on behalf of the Rostock University Medical Center. The authors declare no other competing financial interests.

## Figures and Tables

**Table 1:** Statistical analysis of the Western blot analysis for the *in vitro* evaluation of the antifibrotic effect of kitasamycin (KM) in a TGF-β1-mediated fibrotic cell culture model. Statistical analysis was performed by t-test analysis (two-sample t-test). Untreated fibroblasts (hTFs) and TGF-β1-stimulated fibroblasts (hTFs + TGF-β1) were compared with the culture approaches.

t-Test Analysis (Two-Sample t-Test)		Fibronectin		Collagen VI		Vimentin		α-SMA		β-Actin	
		*p*-Value	Level	*p*-Value	Level	*p*-Value	Level	*p*-Value	Level	*p*-Value	Level
hTF	hTF + 1 µM KM	0.183	n.s	0.178	n.s	0.085	n.s	n.d	-	0.835	n.s
	hTF + 10 µM KM	0.111	n.s	0.117	n.s	0.046	*	n.d	-	0.153	n.s
	hTF + 50 µM KM	0.431	n.s	0.004	**	0.141	n.s	n.d	-	0.200	n.s
	hTF + 100 µM KM	0.000	***	0.234	n.s	0.637	n.s	n.d	-	0.523	n.s
	hTF + TGF-β1	0.000	***	0.090	n.s	0.915	n.s	0.000	***	0.027	*
	hTF + TGF-β1 + 1 µM KM	0.000	***	0.121	n.s	0.466	n.s	0.000	***	0.252	n.s
	hTF + TGF-β1 + 10 µM KM	0.000	***	0.122	n.s	0.006	**	0.000	***	0.127	n.s
	hTF + TGF-β1 + 50 µM KM	0.001	***	0.056	*	0.031	*	0.035	*	0.321	n.s
	hTF + TGF-β1 + 100 µM KM	0.050	*	0.037	*	0.145	n.s	n.d	-	0.154	n.s
hTF + TGF-β1	hTF	0.000	***	0.900	n.s	0.915	n.s	0.000	***	0.027	n.s
	hTF + 1 µM KM	0.000	***	0.013	**	0.069	n.s	0.000	***	0.017	*
	hTF + 10 µM KM	0.000	***	0.015	**	0.054	*	0.000	***	0.514	n.s
	hTF + 50 µM KM	0.000	***	0.001	***	0.149	n.s	0.000	***	0.231	n.s
	hTF + 100 µM KM	0.001	***	0.077	n.s	0.571	n.s	0.000	***	0.471	n.s
	hTF + TGF-β1 + 1 µM KM	0.616	n.s	0.659	n.s	0.414	n.s	0.584	n.s	0.213	n.s
	hTF + TGF-β1 + 10 µM KM	0.533	n.s	0.614	n.s	0.005	**	0.312	n.s	0.107	n.s
	hTF + TGF-β1 + 50 µM KM	0.131	n.s	0.695	n.s	0.028	*	0.001	**	0.518	n.s
	hTF + TGF-β1 + 100 µM KM	0.290	n.s	1.000	n.s	0.131	n.s	0.000	***	0.852	n.s

Not significant: n.s. A *p*-value of ≤0.05 was considered statistically significant. The * symbolizes the significance level: * *p* ≤ 0.5; ** *p* ≤ 0.01; *** *p* ≤ 0.001.

## Data Availability

The data presented in this study are available upon request from the corresponding author.
